# cDNA sequences reveal considerable gene prediction inaccuracy in the *Plasmodium falciparum *genome

**DOI:** 10.1186/1471-2164-8-255

**Published:** 2007-07-27

**Authors:** Fangli Lu, Hongying Jiang, Jinhui Ding, Jianbing Mu, Jesus G Valenzuela, José MC Ribeiro, Xin-zhuan Su

**Affiliations:** 1 Laboratory of Malaria and Vector Research, National Institute of Allergy and Infectious Diseases, National Institutes of Health, Bethesda, Maryland, USA; 2 Department of Parasitology, Zhongshan School of Medicine, Sun Yat‑sen University, Guangzhou, Guangdong 510080, PRoC; 3 Bioinformatics Unit, Laboratory of Neurogenetics, National Institute on Aging, National Institutes of Health, Bethesda, Maryland, USA

## Abstract

**Background:**

The completion of the *Plasmodium falciparum *genome represents a milestone in malaria research. The genome sequence allows for the development of genome-wide approaches such as microarray and proteomics that will greatly facilitate our understanding of the parasite biology and accelerate new drug and vaccine development. Designing and application of these genome-wide assays, however, requires accurate information on gene prediction and genome annotation. Unfortunately, the genes in the parasite genome databases were mostly identified using computer software that could make some erroneous predictions.

**Results:**

We aimed to obtain cDNA sequences to examine the accuracy of gene prediction *in silico*. We constructed cDNA libraries from mixed blood stages of *P. falciparum *parasite using the SMART cDNA library construction technique and generated 17332 high-quality expressed sequence tags (EST), including 2198 from primer-walking experiments. Assembly of our sequence tags produced 2548 contigs and 2671 singletons *versus *5220 contigs and 5910 singletons when our EST were assembled with EST in public databases. Comparison of all the assembled EST/contigs with predicted CDS and genomic sequences in the PlasmoDB database identified 356 genes with predicted coding sequences fully covered by EST, including 85 genes (23.6%) with introns incorrectly predicted. Careful automatic software and manual alignments found an additional 308 genes that have introns different from those predicted, with 152 new introns discovered and 182 introns with sizes or locations different from those predicted. Alternative spliced and antisense transcripts were also detected. Matching cDNA to predicted genes also revealed silent chromosomal regions, mostly at subtelomere regions.

**Conclusion:**

Our data indicated that approximately 24% of the genes in the current databases were predicted incorrectly, although some of these inaccuracies could represent alternatively spliced transcripts, and that more genes than currently predicted have one or more additional introns. It is therefore necessary to annotate the parasite genome with experimental data, although obtaining complete cDNA sequences from this parasite will be a formidable task due to the high AT nature of the genome. This study provides valuable information for genome annotation that will be critical for functional analyses.

## Background

Malaria parasites infect and kill millions of people in the tropics each year [1,2]. Efforts to develop vaccines have so far failed to produce any effective vaccine. Additionally, drug-resistant parasites are spreading quickly, particularly parasites resistant to chloroquine, leading to a recent resurgence of malaria in many developing countries [3,4].

To facilitate our understanding of parasite molecular biology and development of drugs and vaccines, the genome of the malignant human malaria parasite *Plasmodium falciparum *was sequenced and published in 2002 [5]. The genome sequence provides a basis for various genome-wide approaches such as microarray and proteomic analyses [6-9]. Unfortunately, the majority of the genes in the *P. falciparum *genome were predicted using computer software, with ~60% of the predicted genes encoding hypothetical proteins [5]. Although software 'trained' with well characterized genes and improved strategies have provided relatively accurate gene prediction [10,11], the accuracy of gene prediction of this organism is unknown. It is therefore necessary to verify the predictions with complementary DNA (cDNA) sequences, particularly for eukaryotic organisms that have introns in their genes. Indeed, full-length cDNA clones from many species from Drosophila to human have been collected and characterized [12-16], providing important information for verification of genes in a genome and for studying gene functions. Recently, when a high-density array was used to survey transcribed exons, up to 30% of the detected transcripts were found to be unannotatd even in the well characterized Drosophila genome [17].

*P. falciparum *has a unique genome with a very high AT content (~82% of AT) [5] that presents various difficulties for studying gene structure and gene function. The extremely high AT content in non-coding regions (up to 99%) is often an obstacle to obtaining sequences from introns, 5' and 3' untranscribed regions (UTR), and intergene sequences. *P. falciparum *DNA is often unstable in bacteria, making it almost impossible to obtain full cDNA clones from genes larger than 5 kb for expression or other analyses. Approximately 50% of the genes in the *P. falciparum *genome were predicted to have introns flanked by the conserved eukaryotic GT-AG intron-exon splice sites [18,19]. The parasite genome also has many large open reading frames (ORF) that likely encode large transcripts; however, introns imbedded in the ORF cannot be ruled out [20]. The elements regulating gene expression such as promoters and polyA recognition sites seen in other eukaryotic cells may not function properly in this parasite due to the high AT content in noncoding regions [21].

Expressed sequence tags (EST) from malaria parasites, particularly *P. falciparum, *have been obtained previously [19,22-27]. The first survey of *P. falciparum *EST produced 389 tags from 550 random cDNA clones [22]; and the number of EST was later increased to ~2,500 [23]. More recently, 2490 single random sequences were obtained from a library enriched for full-length cDNA [19], which were updated to 11424 sequences covering 1357 predicted genes [27]. cDNA sequences from the full-length cDNA clones (mostly sequences from 5' UTR) identified new genes and multiple transcript initiation sites in some genes, but it appeared that no efforts were made to obtain complete cDNA sequences from full-length cDNA clones. In this report, we constructed various cDNA libraries from mixed blood stages, including three cDNA libraries with different sized inserts enriched for full-length transcripts and sublibraries that contain smaller clones after digestion of the initial inserts with restriction enzymes. We also used synthetic oligonucleotides to extend sequences deep into coding regions. We obtained a total of 17332 clean EST. Comparison of our EST, the EST in public databases, the predicted coding sequences (CDS), and genomic DNA sequences identified 393 genes that may be incorrectly predicted.

## Results and Discussion

### cDNA libraries and DNA sequencing

Collection of EST from *P. falciparum *has been reported previously, and searches of public databases found 21305 *P. falciparum *EST in PlasmoDB [28,29] and GenBank, contributed by various research groups [19,23,27] (Washington University, unpublished). The majority of EST collected previously were short sequences from single sequencing reads. To obtain longer cDNA sequences, we used two different approaches–primer-walking and construction of sublibraries of restriction enzyme-digested DNA clones–to extend sequence reads into the cloned DNA. Three different libraries, each with three sublibraries of different insert sizes, were constructed using polymerase chain reaction (PCR) products after 11 cycles of amplification (Additional file 1A and 1B). The first library contained cDNA clones directly from 5'-enriched cDNA inserts, which were divided into groups of large (> 3 kb), medium (1–3 kb), and small (< 1 kb) insert sizes (Additional file 1B). Unfortunately, we were not able to obtain sequences from either 5' or 3' ends of many clones from this library, probably due to polyA or polyT sequences in non-coding regions, suggesting that these clones may contain full coding sequences. We then constructed sublibraries with DNA inserts digested with restriction enzymes *Bam*HI or Sau3A before cloning into the vector (Additional file 1A).

### Sequence trimming and contig assembly

A total of 28416 sequence runs–including 10656 from 'full-length' libraries, 10368 from *Bam*HI-restricted libraries, 7392 from Sau3A-digested libraries, and 4,800 runs from primer walking–were performed. From the sequence runs, we obtained 17332 EST 100 base pairs (bp) or longer [GenBank EL492722-EL510074] after trimming and vector sequence cleaning (see Methods). Because of difficulty in obtaining sequences from AT-rich sequences in non-coding regions and sequences with polyA tails, most of the sequences were from digested libraries or from the 5' ends of the undigested libraries. The trimmed EST from our libraries were assembled into 2548 contigs and 2671 singletons with an average size of 473.4 bp and an average qual value of 64.7. When our EST were assembled with EST in public databases, we obtained 5220 contigs and 5910 singletons with an average size of 520 bp.

### Genome-wide cDNA coverage

**Figure 1:**
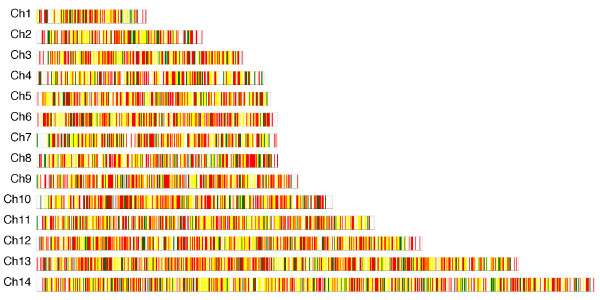
Diagram of the 14 *P. falciparum *chromosomes showing positions of potentially expressed genes. Expressed sequence tags (EST) from our libraries or from public databases were assembled against predicted coding sequences in PlasmoDB; genes that matched our EST only (green), EST already in public databases (red), or both (yellow) are displayed according to gene order on the chromosomes. Those in white are CDS that were not covered by any EST. Approximately 70% of the 5485 predicted CDS were matched with one or more EST.

To determine patterns of genome-wide gene expression and locations of EST on chromosomes, we assembled our EST and the public EST with 5485 predicted CDS in PlasmoDB (version 5.2) and displayed them on the physical chromosomes (Figure 1). When assembled using CAP3 [30] (21 bp overlap and 85% identity), 3857 CDS were assembled with EST contigs. When the sequences were aligned using Blast and methods described previously [31], 3792 CDS were identified by the same EST with cutoff values of at least 100-bp long and 95% identity. The two methods produced almost identical numbers of hits on predicted CDS. This percentage of genes (~70% of total predicted genes) with EST coverage is a little higher than those detected using a 70mer oligonucleotide array (~60%) [6]. Among those EST matching CDS, approximately 42% (or ~1700 genes) were matched by EST from both our collection and those in public databases.

Alignment of cDNA to predicted genes on physical chromosomes allowed us to identify chromosomal regions that are transcriptionally active or silent. Our results show that genes located at telomere or subtelomere regions of many chromosomes (for example, genes at ends of chromosomes 7 and 10) do not have matching cDNA or are largely silent (Figure 1). The chromosome ends of *P. falciparum *are highly variable, consisting of many multigene families such as *rifin*, *stevor*, and *var *[5]. Although the functions of the proteins encoded by *rifin *and *stevor *are still uncertain, the *var *gene family has been shown to encode variant proteins (PfEMP1) that can mediate parasite adhesion to receptors on host endothelial cells [32-34]. Different observations on the expression of the genes at chromosomal ends have been reported using microarray hybridization, with one reporting silent chromosome ends [6] and another suggesting expression of genes from chromosome ends [7]. Because microarray is based on probe-target hybridization, cross hybridization among probes from members of gene families could produce false-positive signals under some hybridization conditions. Our data are consistent with results showing that RNA transcripts from only a small subset of these genes could be detected in intraerythrocytic stages [6]. Additionally, there are regions in the middle of the chromosomes with genes that do not have cDNA coverage (Figure 1).

### Full-length cDNA sequences and discovery of new introns

One of our goals was to collect complete cDNA clones and sequences from the *P. falciparum *genome. Unfortunately, we encountered difficulties in sequencing highly AT-rich regions, mostly 5' and 3' UTR, and obtained only 199 contigs that cover the entire ORF of 87 predicted genes, with predicted ORF sizes ranging from 126 to 2709 bp (Additional file 2). Among the 87 genes, 21 (~24%) were predicted incorrectly (or mismatched), with 18 genes having 23 additional introns and 3 genes with cDNA sequences running into predicted introns. Of the 23 new introns, 21 were found 5' of the predicted ATG, suggesting either additional exons or introns in the predicted non-coding regions. Assembly of our EST and those in public databases increased the number of genes 'fully' covered by EST to 356, with 85 (~24%) genes having mismatched introns (Table 1; Additional file 2). If we assume an error rate of gene prediction for the whole genome similar to that seen in the 356 fully covered genes, we would expect 1316 genes (24% of 5485 genes) being predicted erroneously. This is quite a large number of predicted genes that may have to be re-annotated, which argues for efforts to experimentally annotate the genome using full-length cDNA sequences.

**Table 1:** Predicted coding regions that were covered fully by cDNA and their mismatched introns

Ch	No. genes	Mis intr	New intr	Lost intr	Size change	AS intr
1	11	1	1	0	0	0
2	18	6	7	0	1	1
3	14	4	5	0	0	1
4	14	2	0	1	0	1
5	23	4	5	0	1	0
6	17	3	4	0	0	0
7	14	1	1	0	0	0
8	14	6	7	0	1	1
9	24	4	4	0	1	0
10	23	5	5	0	0	1
11	39	13	16	1	0	1
12	22	2	1	0	1	0
13	61	17	20	1	3	6
14	62	17	24	2	2	2

	356	85	100	5	10	14

Ch, chromosome; No genes, numbers of genes; Mis intr, numbers of genes with introns not matching those predicted; New intr, numbers of new introns found; Lost intr, numbers of introns that may not exist as predicted; Size changes, numbers of introns with sizes that do not match those predicted; AS intr, numbers of introns that may be alternatively spliced. Most known genes are housekeeping genes, consistent with expression profiles.

Approximately half of the *P. falciparum *genes (53.9%) were predicted to contain introns [5]. Our data suggest that the percentage of genes with introns will be higher than the predicted 54%. Among the 21 genes found to have new introns in cDNA, 10 were predicted to have no introns, and one gene predicted to have only one intron actually had none. This represents a net gain of 9 genes with introns among the 87 genes (or ~10%). Among the 85 genes with mismatched introns from the 356 genes with full coverage of predicted coding sequences, 21 genes gained introns (~5.9%), Based on these data, we can predict that about 60% to 65% of the genes in the *P. falciparum *will have one or more introns. Of interest, the majority (> 90%) of the new introns were found at 5' and 3' UTR or within 100 bp from a predicted ATG or stop codon, suggesting additional exons or changes of start or stop codons. It is also possible that the proposed genome sequence contains insertion/deletion errors causing apparent frameshift. Automatic prediction algorithms would then have to find an intron/exon border adding one spurious intron.

Alignment of our cDNA contigs with predicted CDS also identified 78 genes, although not fully covered by our cDNA sequences, with 88 introns either missed by computer prediction or predicted incorrectly (Additional file 3). Among them, 26 genes have 38 introns missed by computer prediction; 25 genes have falsely predicted introns (*i.e*., they do not exist); 22 genes have 25 introns larger than predicted; and 11 genes have 13 introns smaller than predicted. There are also three predicted genes (PFA0175w, PFB0610c, and PFL2160c) that have cDNA sequences extending into their neighboring genes (PFA0180w, PFB0605w, and PFL2155w, respectively). These predicted gene pairs are 200 bp or less apart on the chromosomes. It is likely that the 3' UTR of the genes will be longer than 200 bp, particularly for gene pairs PFB0610c/PFB0605w and PFL2160c/PFL2155w with ORF in opposite orientations. Similarly, assembly of our and public EST with predicted CDS and genomic DNA increased the number of genes having incorrectly predicted introns to 305, with 152 new introns found and 182 introns having sizes different from those predicted (Table 2; Additional file 3). These genes will require further experimental verification with complete cDNA sequences.

**Table 2:** Genes having introns that do not match those predicted in public databases

Ch	No Genes	New intr	Lost intr	Larger intr	Smaller intr	AS intr	Antisense
1	7	4	3	1	3	0	0
2	14	11	3	7	2	4	1
3	10	1	3	2	2	2	0
4	18	2	12	8	4	2	2
5	17	5	3	8	6	2	1
6	18	4	9	10	4	0	0
7	13	6	6	6	3	2	1
8	4	1	1	1	0	0	0
9	3	1	0	3	0	0	0
10	41	18	18	12	12	5	1
11	46	46	10	17	7	3	2
12	37	6	13	10	7	5	4
13	36	3	29	10	8	5	0
14	41	44	12	19	10	9	0

	305	152	122	114	68	39	12

Ch, chromosome; No genes, numbers of genes; New intr, numbers of genes with new introns; Lost intr, numbers of genes with predicted introns, but not confirmed with cDNA; Larger intr, number of genes with introns larger than predicted; Smaller intr, number of genes with introns smaller than predicted; AS intr, introns potentially alternatively spliced. Antisense, numbers of antisense transcripts based on the presence of conserved intron splice sites (GT-AG) in antisense orientation.

### Confirmation of conserved GT-AG intron splicing sites and alternatively spliced introns

All the introns confirmed by our cDNA sequences have typical eukaryotic GT-AG splicing sites except a few genes that have potential 'introns' lacking GT-AG. These atypical 'introns' could be due to deletion during cloning in bacteria. For example, a 497 bp gap was found at 32 bp 5' of the ATG in gene MAL13P1.130, but no GT-AG sites were found in the gap. Gaps without GT-AG sites can be due to either deletion during cloning in bacteria or sequencing errors, although it cannot be ruled out that some introns may not have the conserved GT-AG sites. To investigate this possibility, we designed PCR primers flanking the 497-bp gap in MAL13P1.130 and confirmed the absence of the 497 bp gap (Table 3 and data not shown). Similarly, gene PFL0290w has a gap of 287 bp without GT-AG sites within the predicted ORF; we could not confirm the gap, either. It is clear that gaps without GT-AG sites are unlikely to be true introns. This observation also shows that sequences, including coding regions with relatively high GC content, can be deleted during cloning in bacteria.

**Table 3:** PCR verification of selected introns that were alternatively spliced

Gene ID	Ch	Forward primer	Reversed primer	Gen	Spl	Comments
PFB0260w	2	TCAAACACACGTTACACCT	ATGACAATACCTTCTAAGG	242	102	Confirmed
PFB0305c	2	ACCTTTTGTTAATTATGGA	CCACCTTCTCCTTTTTCG	255	135	Confirmed
PFB0177c	2	ACTAATGGTAGAATAGGTG	TTTCTCCATTTTGTATATCG	373	161	Confirmed
PFB0535w	2	CAAAGATAAAATGGTAATGTT	ATTCCTATTATAGTGTGTGT	507	297/243	No 243-bp band
PFC0371w	3	CCTACCTTCTATTTACAAAT	ACTTGTTGCTCTGATATAAT	301	204	No 204-bp band
PFD0810w	4	GCTGTGAAAAAAGAAAACAA	TTGTTTTCTTTTTTCACAGC	320	174	No products
PFD0895c	4	TTGATAACAATCCTTTAAGC	AATTCGTAATAATCATCTCC	374	205	Confirmed
PFE1540w	5	GATCCTGAAATTGTTTGTG	ATGGCCAAAATGTTTCACA	393	328/283/210	Confirmed
MAL8P1.81	8	GCTGACATATTTATCTTATG	CATATAAGTATTCATGCATG	303	147	Confirmed
PF10_0096	10	ATATTATCGATATTGTCTATATTC	CTTGCTTTGTTTGGCTTCCA	441	182	Confirmed/antisense
PF10_0170	10	TATATTTGTCCTCAGTGC	CTTCCATATCAGATGCCA	300	135	90-bp band, not 135
PF10_0017	10	GGATAAATAGTTTTTTGCTT	CTCAGACAATGTACGCATA	410	263	Confirmed
PF10_0117	10	ATTGGAATTTAACTAGCAAC	TTCATAAGAGTGTTGTTCG	330	134	Confirmed
PF10_0213	10	GGTGCGAATAATAAAGTAG	CTACTTTGTTATTATCTCC	349	229	Add'l 150-bp band
PF10_0247	10	AATTACAAACAATTTGAGGG	TTCATTTTTCAAAAATGCGG	383	152	No 383-bp band
PF10_0258	10	AAAGACGAGGAACTTAATAC	CTCTGATTCTTTTATGAAAG	270	150	Confirmed
PF10_0415	10	CACCAATTTATAAAAGAAGAA	GGCAATAAAAAAGCCTGTTA	370	183	Confirmed
PF11_0292	11	AAGATGACCAACAAGAAGAA	TTATAGTACTCAATAACCTG	340	153	Confirmed
PF11_0377	11	CCGAAAAGGATAAGAAGAAG	TGATTATATGCTGCATATAC	1425	168	No 1425-bp band
PF11_0167	11	TAAGAAATTATGTTCCCAAT	TTTTTCTCCTACACAAGTGC	354	152	Confirmed
PF11_0405	11	TGAACTTAATACACATACGT	ACAGTATCTGAAGGATCTGT	201	130	No 130-bp band
PFL0020w	12	TTCGATATATCATTCCATTC	AAACAGCTACTAGTTGTCC	261	78	No 78-bp band
PFL0290w	12	CTTTATATTATCCAACAACAC	TTGTAATTACTTATAGGAGC	454	167	No 167-bp band
PFL0580w	12	GATGCAATATTAGGTAGACT	ACTAAAGATTAGGTTAACAC	294	193	Confirmed
PFL0890c	12	GAAATGCTCAACAAATTTGA	ACAGATATTATGGGAATTTC	255	130	Confirmed
MAL13P1.130	13	GTATCCAGAAATATTTTTTAC	GTATCAAAAATCCAACACGTA	800	303	No 303-bp band
MAL13P1.183	13	CTCCTAGAAATCCTAGATAT	GACTATGCAGTTTTTTTTATC	315	311	Add'l ~90-bp band
MAL13P1.51	13	CATTTATTGAATGCTCAGC	GTAGTAATATTCTCTCCTG	180	46	Confirmed
MAL13P1.80	13	CCAAAAAAGGACCTAATAAA	TATATATATGCACACGACAT	376	219/150	No 150 bp band
PF13_0082	13	CGAAGTGACAAAAAAAAGGA	CAGAATTTTTCCTATTATCG	294	118	Confirmed
PF13_0224	13	CTGATTTGTTTTTTCAACAAT	GAGTTATCTATTTTTTTAACC	351	152	Confirmed
MAL13P1.195	13	GAAAATGTCTGTCTTGTCAA	GCGTTCATATCGTCAAAAGA	297	179	Confirmed
MAL13P1.253	13	TTTTTACGAACAAAACGGTT	CTTTTGTTTGATCTAATACC	215	117	Confirmed
PF13_0220	13	AGTCATATCAAAAAATAGCT	GTACTTGTCTGATCTTTCTT	284	123/167	Confirmed
PF13_0301	13	AAAAATGAATGGAGTCCAGC	GCTGTTTTTAAATAAAGGGA	243	146	Confirmed
PF14_0434	14	GGATAGAAGAAACTATAACC	ATGCTATCATACTTACTGG	206	104	Confirmed
PF14_0779	14	CCTGATATGCGTGAAATT	TTTTTTCAATATTGTCGTACC	525	90	No 525-bp band
PF14_0338	14	AAAACAAGAATTTATCACGG	GATTCATTCCTGAATGGTCT	727	116	Confirmed
PF14_0488	14	AAAAAAAGGTCTACAAAAGC	TTGTTAAAATATTCCAAGGC	230	92	Confirmed
PF14_0576	14	GCACAATTTGAAAGAAAATT	ACTCGTGATGTAAATTTTCA	629	230	No 629-bp band
PF14_0787	14	CCTTTATTCATATGTGGAAT	GCAAGAGAAAATGGTTTAATAC	585	120	Add'l genomic bands
PF14_0790	14	GAATAGGAAAATATGCCAAG	GAATTATTACTATTCATCAC	239	111	Confirmed

Ch, chromosome; Gen, expected sizes in base pair from genomic DNA; Spl, expected sizes in base pair if introns are spliced out. PFL0020w has a cDNA with 78 bp intron having GT-AG sites inside an ORF, but no spliced band was detected using PCR. Similarly, PFL0290w has a 287 bp gap without GT-AG sites; this gap was not confirmed. Primers for PFE1540 cover two introns, so multiple forms were expected. Indeed, three transcripts of different sizes were found in one of the introns.

**Figure 2:**
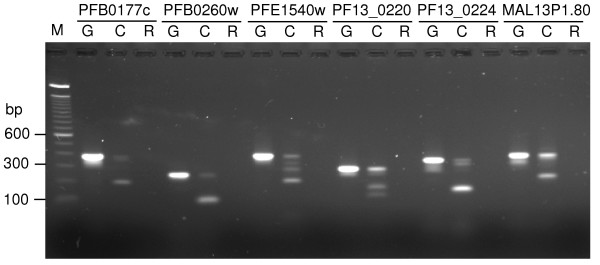
PCR products confirming alternatively spliced introns. Oligonucleotide primers flanking selected predicted introns that might be alternatively spliced were amplified from genomic DNA (G lanes), reverse-transcribed mRNA of mixed asexual stages (C lanes), and mRNA controls of mixed asexual stages (without reverse transcriptase, R lanes). Genes with alternatively spliced introns are as marked; M, 100 bp DNA ladder. Note that more than two bands were amplified from PFE1540w, PF13_0220, and PF13_0224.

Alternative splicing has been well documented in many organisms [35,36] including malaria parasites [37-39]. We noticed that many predicted introns were covered with EST contigs that may or may not have the predicted introns, suggesting potential alternatively spliced introns (Table 3; Additional files 2 and 3), in addition to some cDNA that showed introns of different sizes; however, we could not rule out that those cDNA contigs without introns were from contaminated genomic DNA sequences. To verify these introns, we synthesized primers to amplify some alternatively spliced introns suggested by the cDNA sequences (Table 3). The majority of these introns (except four that have different intron sizes) were either present or absent in sequence alignments, *e.g.*, contigs with some sequences running into the predicted introns. Results from PCR confirmed 29 alternatively spliced introns out of 42 genes tested, including genes with more than two forms of transcripts (Figure 2; Table 3).

### Antisense transcripts

**Figure 3:**
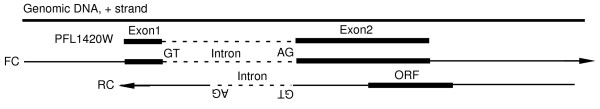
Diagram of exon/intron structures of predicted gene PFL1420w and cDNA contigs covering the gene. FC (forward contig) is a sense transcript with an intron matching the predicted intron. RC (reverse contig) is an antisense transcript having a smaller intron with GT-AG sites in the opposite direction. The line on top represents plus strand genomic DNA. Dashed lines are introns; heavy lines are predicted exons or ORF.

Antisense transcripts are present in the cDNA collections. Because of our cDNA cloning strategies (digestion with restriction enzymes), the orientation of our cDNA clones was not preserved; however, there were transcripts with introns that had conserved GT-AG intron splice sites in the orientation opposite to the predicted genes (Table 2; Additional file 3). These transcripts matched the genomic DNA sequences but with introns having the conserved GT-AG sites in the opposite direction, suggesting antisense transcripts. Of interest, DNA sequence encoding gene PFL1420w (predicted as human macrophage migration inhibitory factor homolog) was matched by two cDNA contigs, one in sense and the other in antisense orientation. The sense sequence had an intron that matches the predicted intron with conserved GT-AG splicing sites. The antisense contig also had an intron with conserved GT-AG sites, but was 121 bp smaller than the predicted sense intron (Figure 3). Translation of the antisense sequence produced a polypeptide with 84 amino acids that had good homology with N-terminal sequence of myosin IXA protein, which could represent a new gene. The presence of these antisense cDNA is consistent with previous reports of antisense transcripts in the parasite [40,41], but the functions of the these transcripts are largely unknown.

### Functional classification

**Figure 4:**
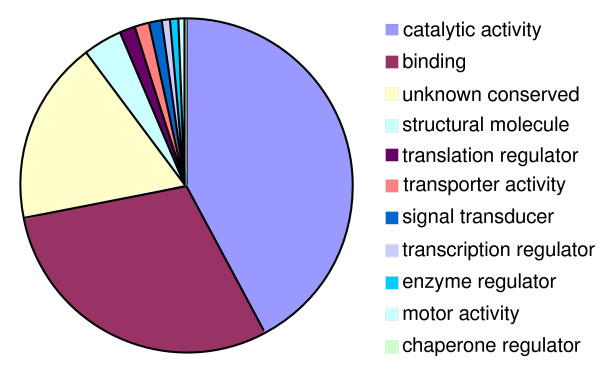
Functional categories of expressed genes covered by all EST. A total of 3862 genes matched by EST were sorted according to GO molecular functions with *P *values < 0.0001 on sequence matches. The majority of the genes encode housekeeping proteins involved in DNA/RNA and protein binding, enzyme catalytic activities, transcription, translation, signal transduction, and transport activities.

The EST contigs matching CDS predictions were grouped as functional categories according to GO molecular functions. As expected, the majority of the genes with functional assignments were housekeeping genes (Figure 4; Additional file 4). Almost all genes with functional assignment among the 356 genes fully covered with EST (likely representing genes relatively small and highly transcribed) were housekeeping genes encoding proteins related to transcription, translation, and other basic cell functions such as ribosomal proteins (41), histone proteins (7), or proteasome proteins (7) (Additional file 2). Based on this observation, we can predict that the majority of the 171 hypothetical genes in Additional file 2 are likely housekeeping genes.

### Potential new genes

There were also contigs and EST sequences that match neither the nuclear genome nor the mitochondrial and plastid genomes (Additional file 4). Some of these sequences might be parasite DNA sequences that were not represented in the finished *P. falciparum *genome. Similarly, there were sequences that match genomic DNA but not the predicted CDS. These sequences could represent new genes or non-coding sequences of intergenic/intron/UTR that require further investigation. For sequence information, linked files and detailed annotation for all the EST contigs, please visit [42].

There are also many predicted ORF larger than 5 kb in the *P. falciparum *genome. The sizes of these large ORF/genes are probably off the limit of cloning stability in bacteria and *in vitro *extension capability of reverse transcriptase. In addition, high AT content in the DNA is an obstacle for obtaining good-quality DNA sequences from PCR products. More efforts with new strategies will be required for obtaining full cDNA sequences for the large genes.

## Conclusion

Although our EST data are still limited, this work obtained 17332 high-quality cDNA sequences that almost double the current EST collection in public databases. Our effort to extend sequences into cDNA clones allows us to assemble some relatively long cDNA sequences and to correct some erroneously predicted introns. Our data suggest that considerably large numbers of genes in this parasite genome may have incorrect intron/exon predictions, arguing for more efforts to collect complete cDNA sequences and reannotate the genome with cDNA sequences. This study also confirms the conserved eukaryotic intron splice site (GT-AG) at the parasite introns, shows the presence of relatively large numbers of alternatively spliced and antisense transcripts, and reveals silence loci at subtelomeric regions of many chromosomes. The cDNA sequences presented here will provide useful resources for genome annotation and analyses of gene expression.

## Methods

### Parasite culture and RNA extraction

*P. falciparum *isolate 3D7 was cultured as described [43,44]. Parasite mRNA was extracted from mixed asexual stages using the Micro-Fast Track mRNA isolation kit (Invitrogen).

### Construction of cDNA libraries

PCR-based cDNA libraries were constructed using a SMART cDNA library kit (BD-Clontech) as previously described [45]. After reversed transcription using polyT primer, the cDNA were amplified for 11 cycles with primers attached to the 5' capping sequences (5'-GCAGTTGTA TCAACGCAGAGTGGCCATTACGGCCGGG-3') and 3' polyT tail. After separation of the PCR products on 1% agarose gel, DNA inserts of large (> 3 kb), medium (1–3 kb), and small (< 1 kb) sizes were eluted from the gel and cloned into Trip-lEX2 vector for trasnfection of XL1blue cells (BD-Clontech). Additional libraries with inserts digested with *Bam*HI and Sau3A were constructed similarly (Additional file 1).

### Sequencing cDNA clones

Plaques were randomly picked and transferred to a 96-well PCR plate (PGC Scientifics) containing 43 μl of SM buffer per well. Each phage sample (5 μl) was used as a template in PCR amplification of the insert using 5' primer PT2F1 (5'-AAGTACTCTAGCATTGTGAGC-3') and 3' primer PT2R1 (5'-CTCTTCGCTATTACGCCAGCTG-3') flanking the cloning sites. For libraries restricted with *Bam*HI or Sau3A, PBKF (5'-ACGGCCAGTGAATTGTAATAC GAC-3') and PBKR (5'-ACAGGAAACAGCTATGACCTTGAT-3') were used in PCR amplification. PCR setups included 30 μl H_2_O, 4.0 μl of 10× buffer, 0.4 μl dNTP (10 mM), 0.15 μl (5 U/μl) Tag polymerase, 0.25 μl of each primer (50 μM), and 5 μl phage solution. The amplification conditions were: 94°C for 5 min; 35 cycles of 94°C for 1 min, 56°C for 10 s, 52°C for 10 s, 60°C for 2 min; and a final extension at 60°C for 5 min. PCR products were treated with 1 μl of ExoSAPIT (United States Biochemical) at 37°C for 15 min and 80°C for another 15 min. Treated PCR products (5 μ) were used in cyclesequencing reaction using BigDye terminator chemistry. The primers for sequencing were PT2F3 (5'-TCTCGGGAAGCGCGCCATTGT-3'), T719 (5'-TAATACGACTCACTATAGGG-3'), or T320 (5'-GAAATTAACCCTCACTAA AG-3'). Sequencing cycles were as follows: denaturing at 94°C for 2 min; 25 cycles at 94°C for 20 s, 52°C for 5 s, 50°C for 5 s, and 60°C for 3 min; and a final extension at 60°C for 5 min. After cleaning with Sephadex 50 beads packed in a multiscreen 96-well cleaning plate (Millipore), the products were analyzed on an ABI3730×l automatic DNA sequencer. To extend the cDNA sequences, 4800 oligonucleotide primers were synthesized based on sequences obtained and used to extend sequences that could not be reached using primers from the vector.

### DNA sequence trimming and assembly

Sequence runs were first base called and assigned quality scores using Phred [46,47] and then trimmed using Lucy [48] to remove sequences shorter than 100 bp or with Phred quality scores lower than 20. Vector sequences and polyA/T were also removed. The trimmed sequences were assembled using CAP3 [30] with 21-bp overlap and 85% identity; the quality of the assembled sequences was inspected visually using Sequencher 4.5 (Gene Codes) and Blast [49]. For sequences having mismatches with predicted CDS (indicating potential incorrect intron/exon predictions), genomic sequences covering the whole predicted coding region plus 1 kb from 5' of start codon and 1 kb from 3' stop codon were downloaded and assembled with EST and CDS. After assembly, the intron/exon junctions were visually inspected and adjusted to ensure proper alignments, particularly for intron splice sites, as software frequently fails to align the A-Trich sequences properly. For *Bam*HI- and Sau3A-digested libraries, some artificial clones from ligation of unrelated DNA fragments were identified and trimmed accordingly after Blast search of the mismatched sequences against the parasite genome sequence.

Locations of each cluster on the assembled chromosomes and the relationships of clusters with each computer-predicted CDS were displayed with Artemis [50]. Sequence annotation, comparison, classification, and functional annotations were performed as described [31] using various software and databases.

## Abbreviations

bp, base pair(s); cDNA, complementary DNA(s); CDS, coding sequence(s); EST, expressed sequence tag(s); ORF, open reading frame(s); PCR, polymerase chain reaction; UTR, untranslated region(s).

## Authors' contributions

FL participated in library construction and in sequencing; HJ and JD participated in sequencing trimming and analysis; JM participated in sequence alignment and PCR; JGV participated in library construction; JMCR participated in sequence clustering, database search, analysis, and manuscript writing; X-z S conceived the project design and participated in analysis and manuscript writing.

## Supplementary Material

Additional file 1 **Construction of cDNA libraries**. The procedures for cDNA librabry construction and sequencing are summarized in diagram (A). PCR products were separated on 1% agarose gel (B) and DNA fragments >3 kb, 1.5–3 kb, and <1.5 kb were eluted from gel blocks. M, molecular weight marker; lane number 8–18 on the gel were products from PCR amplification from 8 to 18 cycles. Eluted DNA fragments were cloned into trip-1EX2 vector that were transfected into bacteria. For construction of sub-libraries, the DNA were first digested with BamH1 or SAU3A and cloned into the same vector. DNA amplified from the vector was sequenced directly. Click here for file

Additional file 2 **Genes with predicted coding regions fully covered by EST and confirmation of predicted introns**. Aligned cDNA and predicted CDS sequences can be viewed by double clicking the hyper-linked gene names in the Excel file. Click here for file

Additional file 3 **Genes with cDNA sequences not matching predicted CDS perfectly**. Aligned cDNA and predicted CDS sequences can be viewed by double clicking the hyper-linked gene names in the Excel file. Click here for file

Additional file 4 **EST contigs that do not match *P. falciparum *CDS and genomic DNA**. For all EST contigs and linked files in additional file 4, please go to [42]. Click here for file
