# Supplementary figures and images for: cDNA sequences reveal considerable gene prediction inaccuracy in the Plasmodium falciparum genome
Source: BMC Genomics. 2007 Jul 27;8:255. doi: 10.1186/1471-2164-8-255 (PMC1978503; doi:10.1186/1471-2164-8-255)

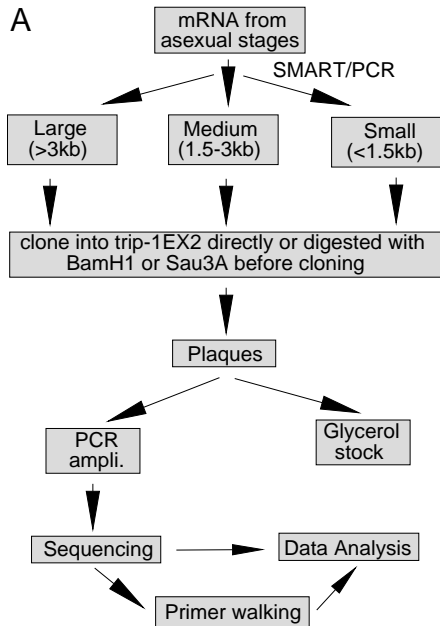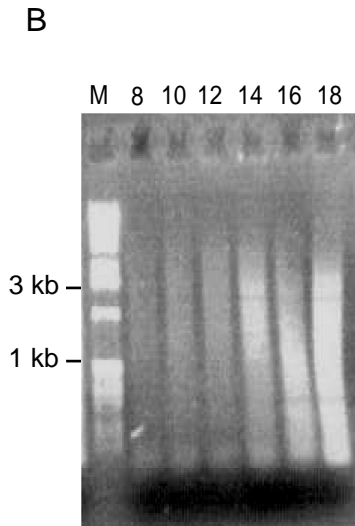

Supplement: Additional file 1 — Construction of cDNA libraries. The procedures for cDNA librabry construction and sequencing are summarized in diagram (A). PCR products were separated on 1% agarose gel (B) and DNA fragments >3 kb, 1.5–3 kb, and <1.5 kb were eluted from gel blocks. M, molecular weight marker; lane number 8–18 on the gel were products from PCR amplification from 8 to 18 cycles. Eluted DNA fragments were cloned into trip-1EX2 vector that were transfected into bacteria. For construction of sub-libraries, the DNA were first digested with BamH1 or SAU3A and cloned into the same vector. DNA amplified from the vector was sequenced directly. [file 1471-2164-8-255-S1.pdf]
